# Global Industrial Development: Insights From the Distribution Dynamics Approach for the Post Covid Era

**DOI:** 10.3389/fpubh.2021.792947

**Published:** 2021-11-23

**Authors:** Ning Ma, Wai Yan Shum, Tingting Han, Tsun Se Cheong

**Affiliations:** ^1^School of Financial Management, Hainan College of Economics and Business, Haikou, China; ^2^Department of Economics and Finance, The Hang Seng University of Hong Kong, Shatin, Hong Kong SAR, China

**Keywords:** COVID-19, industrial development, distribution dynamics, global inequality, convergence

## Abstract

The spread of COVID-19 has significantly dampened global economic activity and has also wreaked havoc on the industrial sector. Understanding the disparity and convergence of global industrial outputs is important in assessing the effectiveness of concurrent development policies. This study investigates the spatial distribution of global industrial output to unveil the disparity in industrial development and the feasibility of achieving convergence over time. Stochastic kernel analyses are carried out for national regimes to study the overall pattern of industrialization for all the countries in the world. Countries are then classified into different groups to further analyse the geographical and income effects on industrial development. The results show that disparity between the Global North and the Global South will enlarge further in the future. Industrial development in the Global North will continue to prosper, while the industrial output in many countries in the Global South just cannot reach the global average.

## Introduction

The World Health Organization (WHO) officially declared the novel coronavirus (COVID-19) outbreak a global pandemic on March 11, 2020. The virus has triggered a series of behavioral and social changes. It is expected that these changes may persist after the pandemic and may have long-term effects on health and productivity (1–3). The coronavirus disease COVID-19 pandemic is caused by severe acute respi-ratory syndrome coronavirus type 2 (SARS-CoV-2) (4). National and local societies around the world are battling the most dramatic global public health emergency of our time, which has quickly become an economic, social, and human crisis that touches all key dimensions of our lives (5). The spread of COVID-19 has significantly dampened global economic activity and has also wreaked havoc on the industrial sector. Recently, numbers of studies have focused on the effects of COVID-19. Some scholars have investigated the effects of COVID-19 on industrial sector (6, 7). The spread of COVID-19 has exacerbated the development inequality of industrial sector. However, studies on disparity and convergence feasibility of industrial development around the globe are limited. This study investigates the spatial distribution of global industrial output to unveil the disparity in industrial development and the feasibility of achieving convergence over time.

Structural issues were once at the core of thinking on economic development policies. Understanding how the industrial development shifts during the development process, affecting the pattern of growth of the economy, has been at the core of economic thinking for decades. The attention has been focused on the process of industrialization as the development of the manufacturing sector has driven the advent of modern economies not only in Europe and North America, but also more recently in East Asia and in Latin America.

Globalization has greatly increased the premium on manufacturing (8). In recent decades, developing countries such as China and India have been able to grow much faster than earlier antecedents (such as Britain and the United States). This is due to the world markets provide near limitless demand for manufactured exports from developing countries. However, the industrial development inequality still exists in the world. Understanding the disparity and convergence of global industrial outputs is important in assessing the effectiveness of concurrent development policies.

There are several gaps in the literature. First, the existing studies on the convergence of industrial output, such as Beyer and Hassel (9), Jefferson et al. (10) and Cheong and Wu (11) still used single country data for the analysis. Neither reveal the industrial development in a global scale. Second, regression models were employed in many previous studies which analyse the impacts of different independent variables on the industrial value added and industrialization (12–14). However, due to the problems of multicollinearity, a major shortcoming is that such regression models cannot include many independent variables. This would cause many of other relevant factors are neglected. Also, the output of regression models is the forecasted value of the dependent variable. This cannot be used to forecast the evolution of a distribution. Moreover, regression models fail to provide information on the value of the convergence and the number of the convergence clubs in the distribution (15). However, this information is important for policy makers.

Many studies have investigated the nature of industrial development and impact of industrialization (13). However, there is limited concerns has been dedicated to whether differences in industrial development among different countries vanish over time, and if convergence can be realized. This study contributes to the existing literature from three perspectives. First, it uses a new stochastic kernel approach in the transition dynamics analysis, which provides an in-depth understanding of the disparity and convergence feasibility of industrial development around the globe. Second, this study utilizes a new framework to analyse the upward development mobility of countries; this mobility analysis helps reveal the mechanism behind the disparity and the convergence. Third, the distribution dynamics and convergence patterns for countries in the Global North and Global South and in different income and geographical regimes are analyzed individually; this helps us to understand the geographical and income effects on industrial development. The findings foster a better understanding of the role of industrialization and provide relevant information for formulating industrial policies.

Policy maker would find this research provide rich policy implications. A comprehensive study on the distribution of the Relative Industrial Value-Added per Capita (RIVAPC) of global countries provide policy makers with reliable reference to improve global industrial development strategies by prioritizing supportive policies across the countries. The world organization for industrial development can encourage capital and technology resources directed to these lagging countries with imbalance RIVAPC. Moreover, the distribution dynamics approach would forecast on the future pattern of country-level RIVAPC, this will show the governments an effective way to promote the rationalization of RIVAPC transition.

The rest of the paper begins with a brief review of the literature in Literature review. Data and Methodology describes the data and methodology. Discussions investigates the dynamics of the spatial distribution of RIVAPC, while Conclusions and Implications summarizes the findings and discusses the policy implications.

## Literature Review

In recent decades, industrial development, industrial output, and industrialization have become the hot topics in the literature. In terms of a global scale, Romano and Traù (16) has used a sample of 63 countries covering the period 1989–2011 to discuss the relationship between industrial development and structural change. They found that industrial development of South of the world might slow down in the future. Xing et al. (17) used GIVCN-WIOT models based on World Input–Output Database to measure the structural indicators of the social and economic system. Fan and Liu (18) used a multi-regional input-output table to estimate the pattern of global industrial trade. Fan and Liu found that in China, the scale of manufacturing relocation has slowed significant; extractive industry has begun to shift to the US and the EU. Landa-Arroyo (19) used international input-output table to identify the position of each country-sector in global value chains. The author found that industrial policies have the significantly effect in Pacific Alliance economies. Although there are only limited studies covers those topics in a global scale in the literature, the authors will still list the relevant literatures based on the large economies in the world below.

Andreoni and Tregenna (20) has evaluated the industrial policy implications for countries such as China, Brazil and South Africa and they found that industrial policy implications for those middle-income countries more widely. Ciccarelli et al. (21) used a historical dataset with annual 1861–1913 data on regional railways endowment and manufacturing value-added to evaluate the relationship between early diffusion of railways and industrial growth in Italy's regions. The result revealed that the contribution of railway developments on industrial development was increasing over time. Liu et al. (22) used Chinese industrial sector data from 2005 to 2016 to study how Artificial Intelligence (AI) affects carbon intensity. They found that AI has significantly reduced carbon intensity in Chinese industrial sector. Tsai (23) has applied Markov regime switching mechanism to evaluate the dynamics of industrial development and structural changes in Taiwan. The results suggested that to improve industrial competitiveness, industries should adopt more sustainable practices. Yuan et al. (24) evaluated the inclusive and sustainable industrial development for 30 provinces in China, the data covers the period from 2011 to 2016. They found that Beijing and Tianjin are the benchmark provinces for promoting inclusive and sustainable industrial development. Liu et al. (25) compared the sustainable development of industrial park between China and Canada and the authors found that industrial parks in Canada appear to be moving more slowly in their adoption of sustainable development. Guo et al. (26) used panel data from 2003 to 2016 studied the relationship between industrial agglomeration and green development efficiency in China and they found that industrial agglomeration has promoted green development efficiency. Zhu et al. (27) proposed a novel integrated approach, taking the provincial data of China from 1999 to 2016 as an example, explored the effect of industrial structure adjustment on green development efficiency. The results revealed that the industrial structure has great impact on green development efficiency. Basakha et al. (28) used econometric models with annual data from 1967 to 2015 and they evaluated the relationship between industrial development and social welfare in Iran. The result revealed that industrial development had a significant impact on the Iranian social welfare and this impact has been stronger in the long run. Nguyan and Ye (29) evaluated sustainable industrial development in the Mekong Delta in Vietnam. They found that industrial development in the Mekong Delta is unsustainable. Tian et al. (30) analyzed the impact of industrial structure change on CO_2_ emission in southwest China during period of 2002–2012. The results indicated that diversification in development and competitive industries had different impacts on CO_2_ emission trends.

Convergence in industrialization has attracted many attentions and most of those studies have focused on China. Arrighi (31) demonstrates empirically that widespread convergence in the degree of industrialization between former First and Third World countries over the past four decades. Kohsaka (32) studied the industrial sector's labor productivity convergence in East Asia countries. Yu et al., (33) found that beta-conditional convergence exists across the carbon intensities of all 24 industrial sectors in China by considering capital intensity and per-capita sectoral value added. In terms of industrialization and inequality, Kuznets (34) suggests that industrialization will lead to an increase in inequality in the early stages of economic development. His argument is widely accepted, and many scholars have studied the relationship between China's industrialization and regional inequality (12, 35–41). Those studies all confirm that industrialization in different countries is positively correlated with inequality.

Other scholars have focused on the study of industrial output inequality and its impact on overall regional inequality, and China has attracted enough attention on this issue. For example, Huang et al. (42) used China's provincial data and performed a decomposition of the Gini coefficient. They found that the inequality in the secondary industry sector was the main contributor to the inequality in aggregate economic development. Cheong and Wu (43) also found that the inequality in the secondary industry sector was the principal contributor to regional inequality in China. Wei (44) examined regional inequality of industrial output in China from 1952 to 1990. The author found that interregional inequality has gradually increased since 1978, but the interprovincial inequality decreased.

Although the inequality in industrial output is worth to investigate, no research has been conducted on the distribution dynamics of industrial output in a global scale. In terms of methodology, σ-convergence and β-convergence are two popular technologies applied in the economic growth studies (45). σ-convergence indicates that the dispersion of real per capita income across countries tends to fall over time while β-convergence applies if a poor country or region tends to grow faster than a rich one (46). However, σ-convergence and β-convergence not been applied to industrial development literature. Recently, such as LM and RALS-LM unit root test have used to test for stochastic convergence (47, 48). However, Quah (49) concluded that these parametric studies are often misleading due to wrong assumptions about the distribution and could not provide the much-needed information of the entire shape of the distribution and its changes. In fact, there are some scholars applied a distribution dynamic approach in studying convergence of different economic characteristics. For example, He (50) investigated the Chinese agricultural sector. Herrerias (51) studied the regional growth in China. Sakamoto and Fan (52) evaluated the regional income disparity in China. Villaverde and Maza (53) studied the Chinese per capita income distribution. Liu and Zuo (54) also studied the dynamics of income distribution in China.

The distribution dynamics is making no assumptions about the underlaying distribution of the population and it allow us to understand the transitional dynamics of RIVAPC over time. This method could provide the intra–distribution mobility for the countries. It can even offer detailed information on each spatial grouping by revealing their distinguishing features in terms of RIVAPC (55). In addition, this approach can be used to offer a forecast for the shape of the distribution of RIVAPC in the long run, which is an issue particularly relevant from the policy implications perspective.

In summary, parametric methods has always been used in the existing literature and there has been no application of a global scale. It is demonstrated below that a distribution dynamic approach which does not assume the underlying distribution of the population has some advantages over the parametric methods. By analyzing the convergence and distribution dynamics of RIVAPC in a global scale and whether such convergence process occurs within a group of regions may show the government an effective way to promote the rationalization of RIVAPC transition.

We try to bridge the gap between the different strands of literature, uses a new stochastic kernel approach in the transition dynamics analysis to estimate the disparity and convergence feasibility of industrial development around the globe. The COVID-19 could exacerbate the inequality in global industrial development and this study has important enlightenment to the Post Covid Era.

## Data and Methodology

The data of the study were obtained from the World Development Indicators provided by the World Bank. The data of value added (constant 2010 US$) for industry (including construction) of each country were collected with its population data. The data cover the time from 2000 to 2017. Frist, the value of value added for industry per capita was computed for each country. The global average of each year was then computed by taking an average of all the value added for industry per capita of all the countries in that year, and the Relative Industrial Value-Added per Capita (RIVAPC) was derived by dividing the data of value added for industry per capita by the average. Almost all the countries in the World Bank database were included in the analysis, though a few countries were excluded due to data unavailability.

This study is based on RIVAPC for each country and distribution dynamics approach was employed to investigate the changes in the distribution and the future steady state distribution in the long run. Moreover, probability of moving upward was also computed for the entities so that one can know about the future development path of the countries and the underlying tread behind the changes in the distribution.

Quah (49) first suggested the use of distribution dynamics approach in his paper in the 1990s. After that, many scholars have employed this innovative analytical technique in their research. This approach can be broadly divided into two categories, the first is called the Markov transition matrix analysis, and the other one is the stochastic kernel analysis. The former has attracted a lot of criticisms as the demarcation of state in the grid selection process is arbitrary. In fact, the analytical results may be affected strongly by this process. Therefore, the stochastic kernel approach is adopted in this study. It is an improved version of the former and the grid selection process can be performed objectively.

The formula of the bivariate kernel estimator is defined as below:


(1) $$\begin{array}{r} \hat{f}\left(x,y\right)=\;\frac{1}{nh_{1}h_{2}}\;\overset{n}{\underset{i=1}{\sum }}K\left(\frac{x-X_{i,t}}{h_{1}},\frac{y-X_{i,t+1}}{h_{2}}\right) \end{array}$$

where *h*_1_ and *h*_2_ are values of bandwidth which were computed by following the approach proposed by Silverman (56), *K* is the normal density function, *n* is the total number of observations in the database, *X*_*i,t*_ is an observed value of RIVAPC at time t, *x* is RIVAPC at time t, *X*_*i,t*+1_ is the observed value of RIVAPC at time t+1, and *y* is RIVAPC at time t+1.

It is worth noting that the data are not evenly distributed for many economic measurements, so a lot of observations may cluster around some specific values, while other values may have only a few observations. Therefore, a two-step procedure suggested by Silverman (56) was used to take the sparseness of the data into consideration. This procedure is called the adaptive kernel and the first step of the process is to compute a pilot estimate for the model, while the second step is to rescale the bandwidth according to the sparseness of the data.

Following the general practice of other distribution dynamics analysis, and assuming first order, and time invariant for the evolution trend. The formula of the relationship between the RIVAPC at time t and time t+1 is:


(2) $$\begin{array}{r} f_{t+\tau }\left(z\right)=\;\int_{0}^{\infty }g_{\tau }\left(z|x\right)f_{t}\left(x\right)dx \end{array}$$

where *f*_*t*_(*x*) is the kernel density function of the distribution of RIVAPC at time t, *g*_τ_(*z*|*x*) is the probability kernel which maps the distribution from time t to t +τ, and *f*_*t*+τ_(*z*) is theτ-period-ahead density function of *z* conditional on *x*.

The ergodic distribution is the steady-state distribution in the long run and it can be estimated by:


(3) $$\begin{array}{r} f_{\infty }\left(z\right)=\;\int_{0}^{\infty }g_{\tau }\left(z|x\right)f_{\infty }\left(x\right)dx \end{array}$$

where *f*_∞_(*z*) is the ergodic density function when τ is infinite. The ergodic distribution is a forecast into the future and it can reveal the final distribution given that the distribution dynamics remain unchanged.

It is worth noting that the probability of the movement of the entities is difficult to observe by naked eye. Therefore, Cheong and Wu (11) developed a new technique, namely the Mobility Probability Plot (MPP) for studying the probability of future movement of the entities. After the invention of this technique, it has been applied widely in many different areas, including industrial output (11) and consumption of electricity (55).

The MPP is defined as *p*(*x*) which is the net upward mobility probability:


(4) $$\begin{array}{r} p\left(x\right)=\int_{x}^{\infty }g_{\tau }\left(z|x\right)dz-\;\int_{0}^{x}g_{\tau }\left(z|x\right)dz \end{array}$$

The MPP plots the net upward mobility probability against RIVAPC. It is expressed in percentage term and so the value is from −100 to 100. A positive value of MPP suggests that the country will move upward and so it will have a higher RIVAPC in the next period, whilst, a negative value of MPP implies that the country has a net probability of moving downward in the distribution and will have a lower RIVAPC in the next period [please refer to (11) for details].

## Discussions

In this section, the distribution dynamics analysis on global industrial development will be presented. The findings can reveal overall pattern of industrialization for all the countries in the world, thereby unveiling the disparity in industrial development and the feasibility of achieving convergence over time. However, in order to investigate this important issue in further details, the full dataset was separated into different groupings. Distribution dynamics analyses were conducted separately for each of these groupings so as to provide an in-depth analysis. The groupings are based on the Global North and Global South, levels of income, and geographical locations.

### All Countries

**Figure 1:**
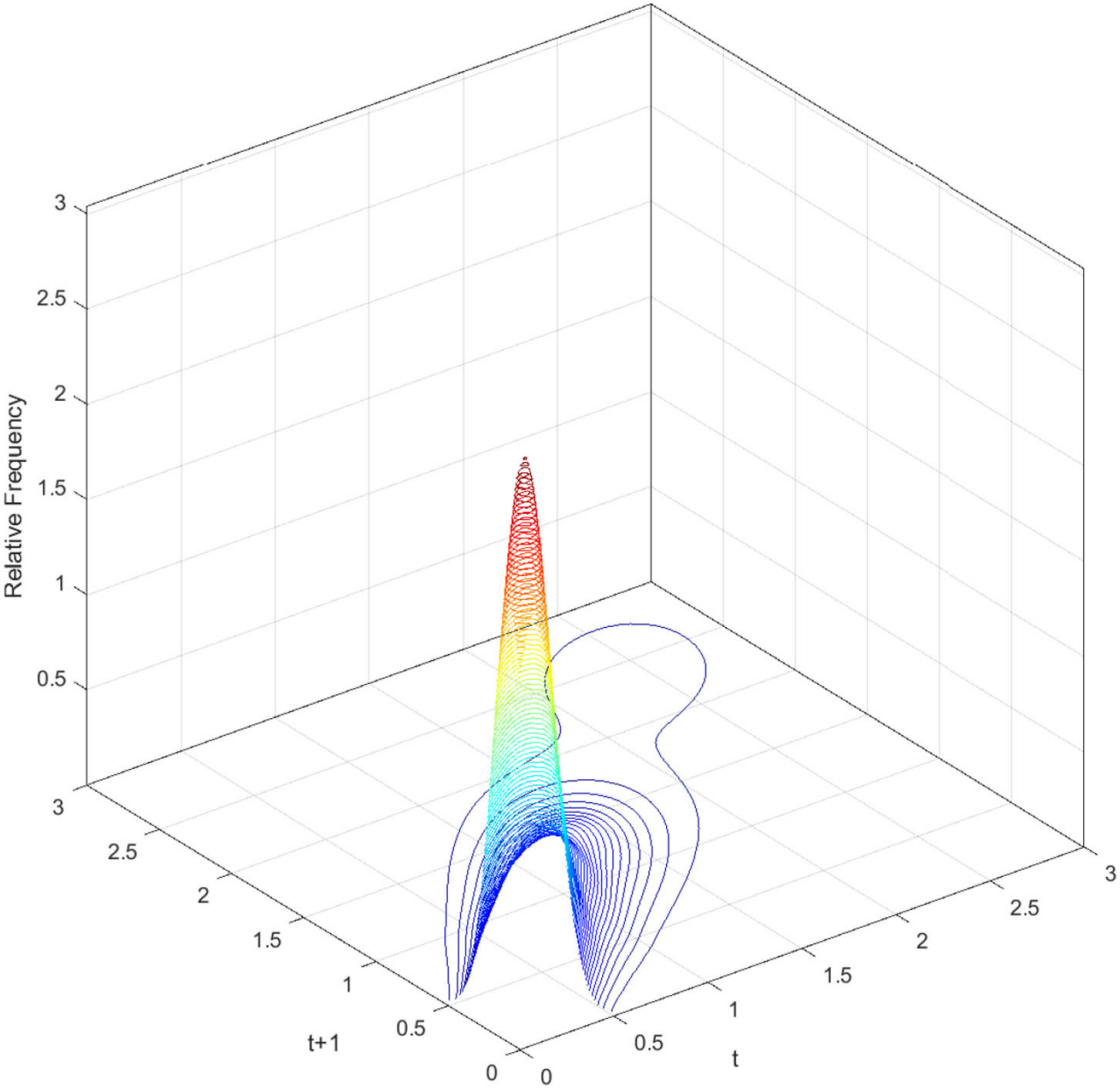
Three-dimensional plot of transition probability kernel for the relative RIVAPC of all countries with annual transitions. Source: authors' calculation. N.B. The vertical axis indicates the probability of transition at the country level from one particular RIVAPC value in year *t* to another RIVAPC value in year *t* + 1.

**Figure 2:**
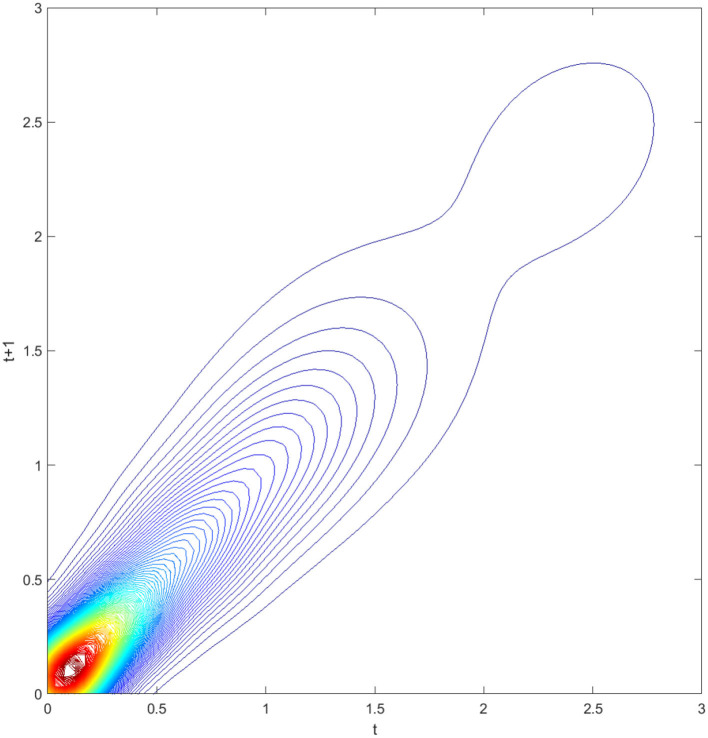
Contour map of transition probability kernel for the RIVAPC of all countries with annual transitions. Source: authors' calculation.

The three-dimensional kernel-based transition probability for the Relative Industrial Value-Added per Capita (RIVAPC) of all countries is demonstrated in Figure 1. Along with the transition dynamics, a contour map of the national units is presented in Figure 2. The relative frequency—that is, the height of the three-dimensional graph—in Figure 1 shows the probability of transition at the country level from one specific RIVAPC value in year *t* to another RIVAPC value in year *t*+1. Note that the RIVAPC is measured relative to the global average; hence, the average of the RIVAPC is one. It follows that a value less than one indicates a below-average RIVAPC, whereas a value larger than one implies that the value is above average. In Figure 1, two different probability mass concentrations of the transition probability are shown; the tallest peak appears at around the RIVAPC value of 0.2, and the secondary peak appears at around the value of 2.5. This pattern of concentration indicates that most countries have a below-average RIVAPC, whereas a small group of countries have a high industrial output. Consequently, it is concluded that there is a noticeable imbalance in industrial development worldwide. This imbalance suggests that most countries possess an extremely low level of RIVAPC.

The contour map in Figure 2 provides a top view of the three-dimensional graph. Thus, each vertical intersection of Figure 2 at period *t* denotes a probability density function that shows the transition probabilities of going from a particular RIVAPC value at period *t* to another value at period *t* + 1. For countries situated on the diagonal line, the RIVAPC levels will be the same before and after transitions. In this regard, first, the higher the concentration of the probability mass along the diagonal line, the higher the probability of continuing in the status quo. Second, the higher the concentration of probability mass above the diagonal line, the higher the probability of upward movement in the next period. Third, the higher the concentration of probability mass below the diagonal line, the higher the probability of downward movement in the next period. Likewise, it can also be asserted that the more dispersed the probability mass, the higher the variability, and vice versa. Figure 2 shows that the peaks of the probability mass lie along the diagonal line; however, the variability is still quite pronounced. Moreover, perseverance is more severe for countries with relatively low or relatively high RIVAPCs, as the variability in probability mass is lower for countries with a RIVAPC less than half of the global average or more than twice the global average. Hence, it is clear from Figures 1, 2 that the progress of industrial development was sluggish for countries with extremely high and extremely low RIVAPC values, thereby suggesting that global industrial patterns are uneven. The disparity and rigidity inherent in the transition dynamics in Figures 1, 2 will eventually translate into a country-level long-run steady-state RIVAPC distribution.

**Figure 3:**
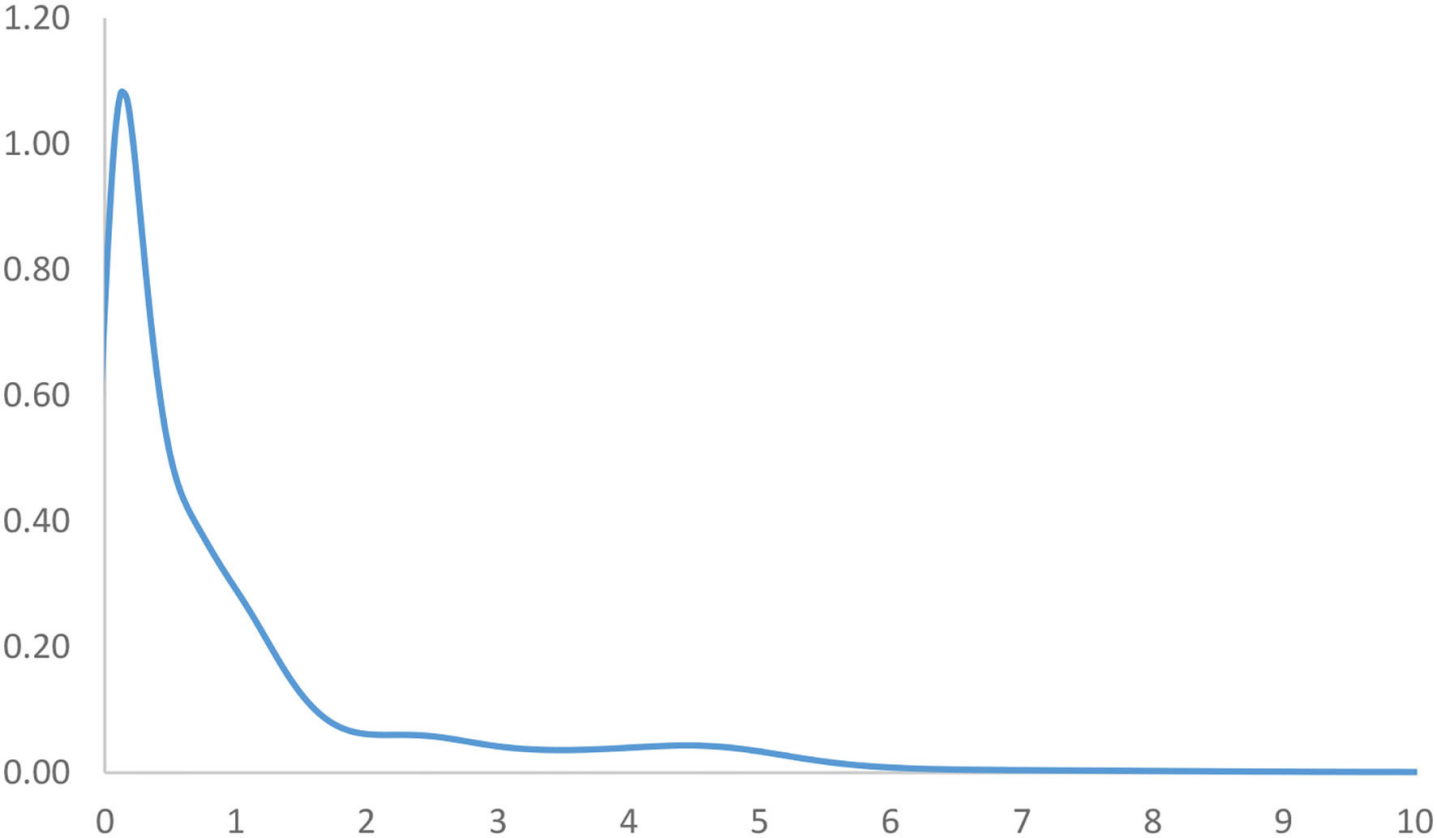
Ergodic distribution for the relative RIVAPC of all countries with annual transitions. Source: authors' calculation. N.B. The vertical axis indicates the density of probability and the horizontal axis indicates RIVAPC values.

The long-run steady-state ergodic distribution of the countries is shown in Figure 3. It can be observed that many countries will converge toward a RIVAPC value of 0.17—the highest peak that can be observed from the distribution. The global average is one; this means that many countries will have very low industrial development if the transition dynamics remain unchanged. Convergence clubs at higher levels can be found as two minor peaks can be observed. These countries will congregate at the RIVAPC values of 2.4 and 4.5, respectively. Although these RIVAPC values are higher than the global average, this will not make the situation more impressive, as it suggests that low production capacity will become the norm for countries worldwide with few exceptions.

Given that industrial development is vital for promoting economic growth, the United Nations and the governments should provide more support to those underdeveloped countries in a more targeted manner through technical co-operation with developing countries. With regard to the issues of low and uneven production capacity, the ideal overall situation would be a gradual convergence to the global average. For this, below-average countries will be required to move upwards and/or above-average ones to move downwards. However, it is undesirable for high-production countries to reduce their output; therefore, more attention should be paid to those countries that are below average, particularly those that do not have the capability of moving up. This study helps identify these countries by looking at the MPP.

**Figure 4:**
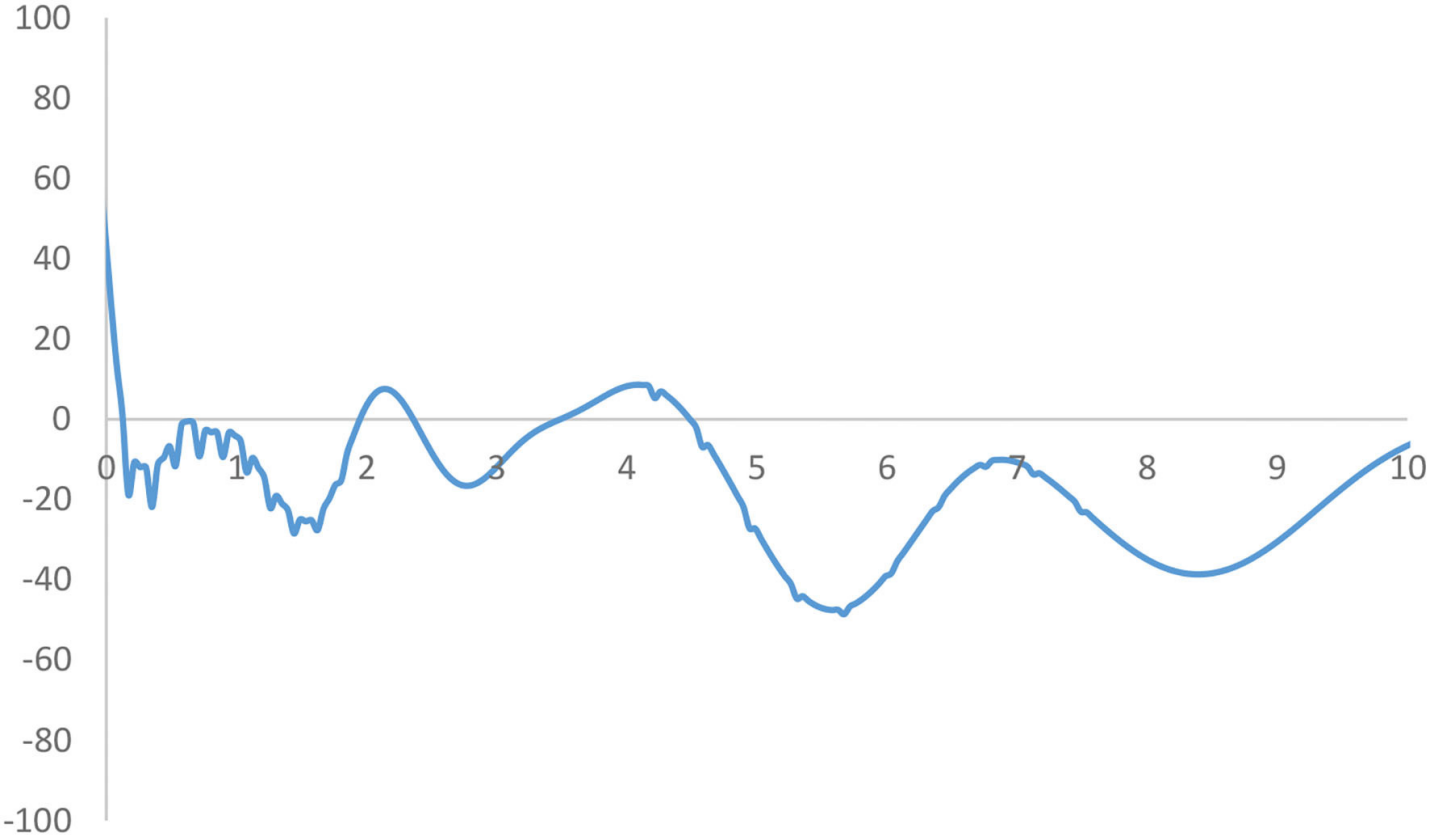
Mobility probability plot (MPP) for the RIVAPC of all countries. Source: authors' calculation. N.B. The vertical axis indicates net upward mobility (%) and the horizontal axis indicates RIVAPC values.

The MPP in Figure 4 plots the probability of net upward mobility as a percentage against the values of RIVAPC. The net upward mobility ranges from −100 to 100; a positive value denotes that the country has a positive net probability of moving upward, while a negative value indicates that a country has a negative probability of moving up. It can be observed that the MPP intersects at the horizontal axis at the RIVAPC value of 0.12; after that, the MPP remains negative (or nearly zero with the RIVAPC values around 0.7) until it intersects the horizontal axis again at the RIVAPC value of 1.95. Thus, among countries which had below-average industrial development, that is, those with a RIVAPC less than one, only those countries which had extremely low output levels (i.e., a RIVAPC < 0.12) had a positive chance to move upward within the distribution. Conversely, countries with slightly higher output levels (i.e., a RIVAPC >0.12) cannot maintain their progress in industrial development. This finding is alarming and suggests that more support should be provided to this group of countries to promote industrialization and provide growth opportunities to people living in these underdeveloped countries.

The MPP will intersect the horizontal axis whenever it moves from above the horizontal axis to the region below the horizontal axis. It is worth noting that the countries on the left-hand side of the intersection point have a positive chance to move upwards and countries on the right-hand side of the intersection point have a net probability of moving downwards; hence, many countries will converge around the intersection points. Consequently, the shape of the ergodic distribution in Figure 3 is explicable through the transition dynamics underlying the MPP in Figure 4.

### Comparison Between the Global North and the Global South

**Figure 5:**
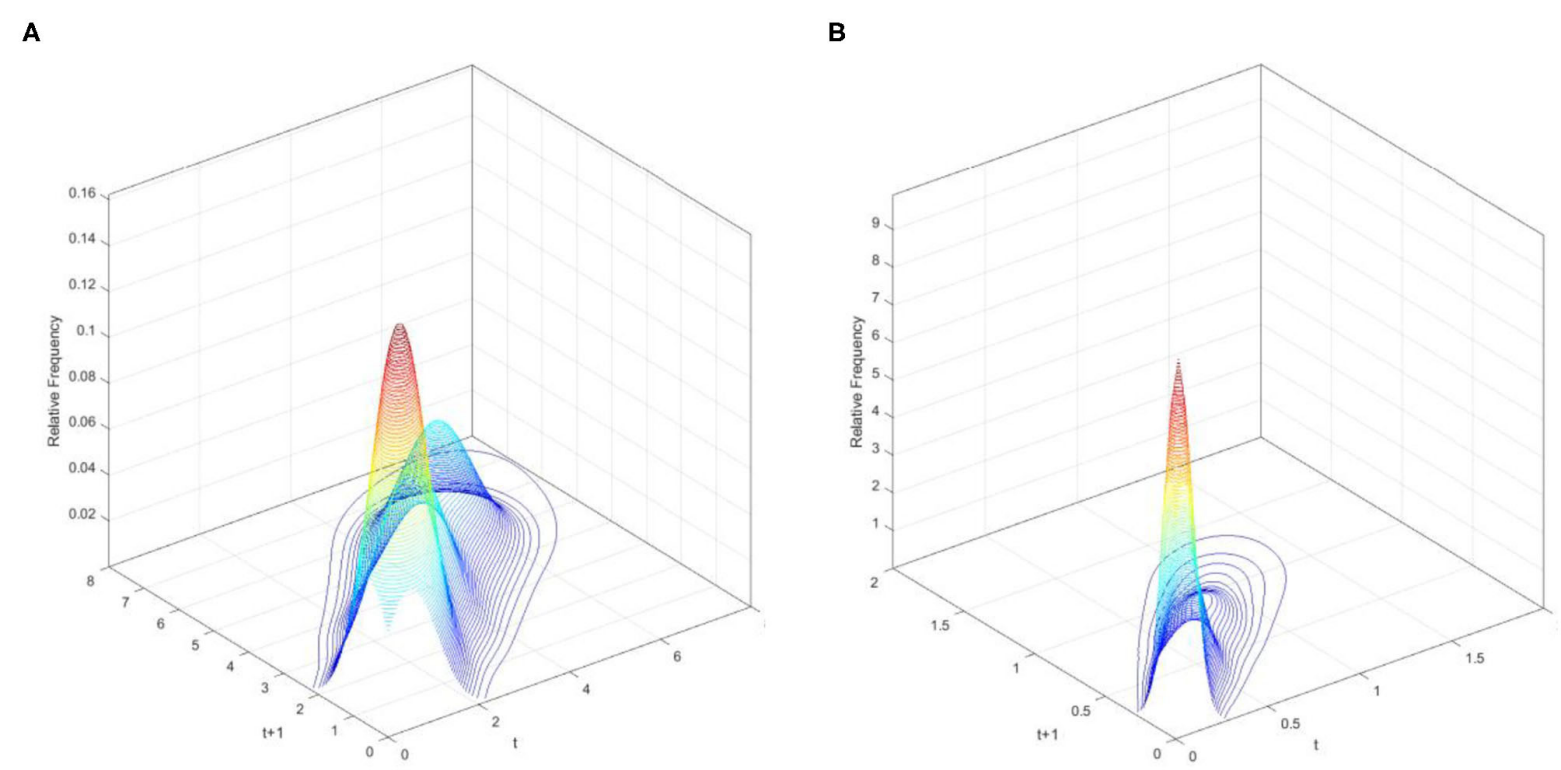
Three-dimensional plot of transition probability kernel for the relative RIVAPC of countries for the Global North and the Global South with annual transitions. Source: authors' calculation. N.B. The vertical axis indicates the probability of transition at the country level from one RIVAPC value in year *t* to another RIVAPC value in year *t* + 1. **(A)** Global North. **(B)** Global South.

**Figure 6:**
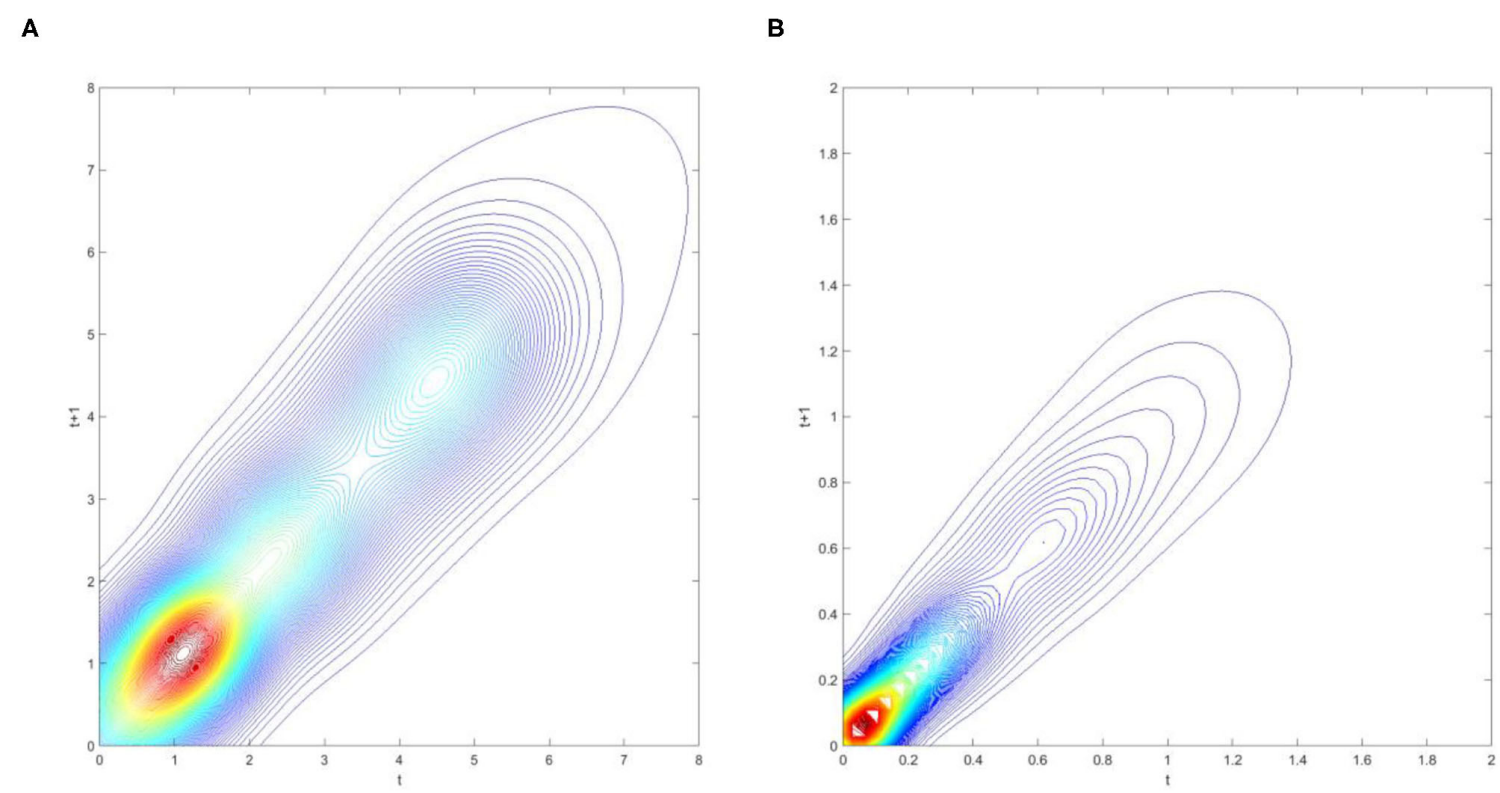
Contour map of transition probability kernel for the RIVAPC of countries for the Global North and the Global South with annual transitions. Source: authors' calculation. N.B. The vertical axis indicates the RIVAPC value in year *t* and the horizontal axis indicates RIVAPC value in year *t* + 1. **(A)** Global North. **(B)** Global South.

To extensively explore industrial growth, the database is split into the wealthier Global North and the poorer Global South to analyse the impacts on industrial development. The three-dimensional kernel-based transition probabilities for the RIVAPC of the Global North and the Global South with annual transitions are shown in Figure 5, and the corresponding contour maps are shown in Figure 6.

Figure 5A demonstrates two peaks in the three-dimensional graph for the Global North. One is situated at a RIVAPC value of 1.25, while the other is situated at around the level of 4.5, suggesting that industrial development for most of the countries within the Global North was well above the global average with a discrete group of countries enjoying 4.5 times above the average capacity for production. Additionally, Figure 5B shows a contrasting scenario. The two peaks: one situated at an extremely low RIVAPC value of 0.05, and the other situated at 0.62, both are far below the global average. This indicates that most of the countries within the Global South suffered from severe underdevelopment, with a few exceptions. As such, the degree of industrial development globally is very unbalanced.

Figures 6A,B show that the variability (i.e., the likelihood of moving upward/downward in the following period) is larger in the Global North than in the Global South. Moreover, variability increases with the increase in production capacity in both the Global North and the Global South. Additionally, observations can hardly be found above the RIVAPC value of 2 in the Global South, indicating that the rigidity at around this value of 2 in Figure 2 is contributed to by countries with relatively higher RIVAPC values. This evidence, together with the evidence in Figure 2, suggests that rigidity is more pronounced in countries with extremely high and extremely low RIVAPC values, which is a potential source of unbalanced industrial development.

**Figure 7:**
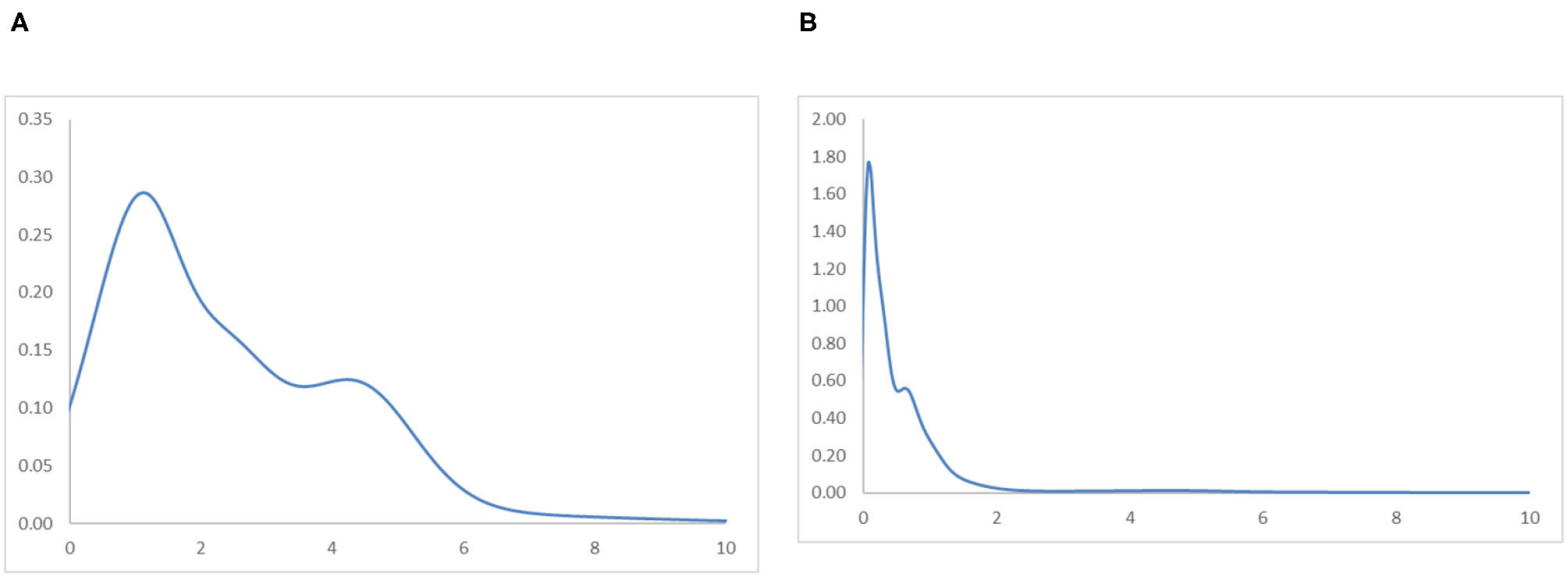
Ergodic distributions for the RIVAPC of countries for the Global North and the Global South with annual transitions. Source: authors' calculation. N.B. The vertical axis indicates the density of probability and the horizontal axis indicates RIVAPC values. **(A)** Global North. **(B)** Global South.

The transition dynamics translate into the long-run steady-state ergodic distribution shown in Figure 7. From Figure 7A, it is visible that the two most obvious peaks appear at around the RIVAPC value of 1.2 and the 4.5 levels, implying that future industrial development in the Global North is very encouraging. Meanwhile, Figure 7B shows that the two peaks are situated at levels of 0.06 and 0.7, respectively. This is a disturbing finding, as it indicates that the Global South countries will have exceptionally low RIVAPC levels in the future. Moreover, the ergodic distribution is more dispersed in the Global North than in the Global South; this implies that countries are highly tied and concentrated around the two below-the -average peaks with very few exceptions in the Global South.

**Figure 8:**
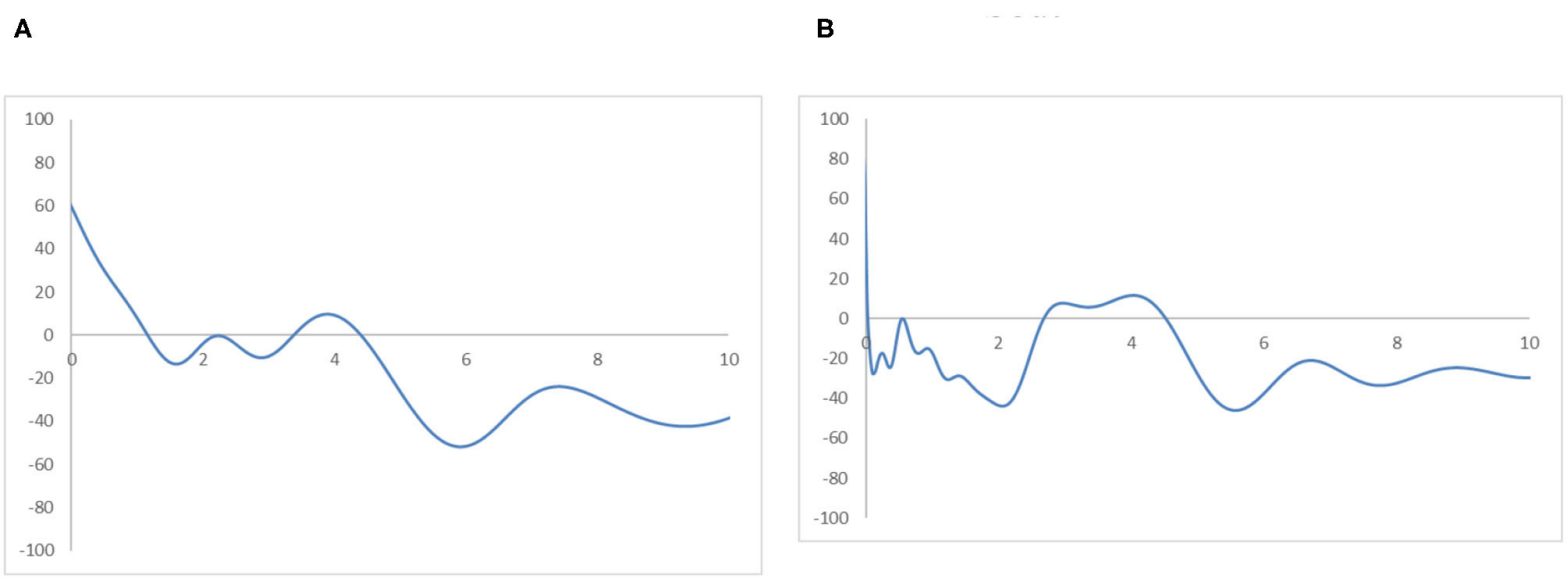
Mobility probability plot (MPP) for the RIVAPC of countries for the Global North and the Global South. Source: authors' calculation. N.B. The vertical axis indicates net upward mobility (%) and the horizontal axis indicates RIVAPC values. **(A)** Global North. **(B)** Global South.

Looking at the MPP in Figure 8, it is clear why the ergodic distributions take their current forms. Figure 8A shows that countries in the Global North with a RIVAPC <1.2 have a positive net probability of moving upwards. The first peak in the ergodic distribution is evident at this intersection. The second intersection point presents at a RIVAPC level of 3.5, and it indicates the secondary peak in the ergodic distribution. These two peaks are above the global average. Thus, the MPP demonstrates how the transition dynamics drive high industrial development in the Global North region. Conversely, almost the entire MPP in Figure 8B is below the horizontal axis. Note that the net probability of moving upward is negative below the horizontal axis. The first intersection point appears at around a RIVAPC value of 0.07, which is far below the first intersection point in the Global North (i.e., RIVAPC value of 1.2). Although, it is visible that a secondary intersection point appears at around the 2.7 level. However, this does not lead to the appearance of another peak in the ergodic distribution. The reason behind this myth is that, in the Global South, a few countries (e.g., China) have a high production capacity and high upward mobility. However, these sporadic cases offer less help in the development of the overall industrial growth in the Global South region. If the sporadic cases in the Global South region are ignored, the entire MPP of the Global South region is below the horizontal axis, except for those countries with a RIVAPC value <0.07. It follows that if and only if countries in the Global South have no industrialization at all (those countries with a RIVAPC value <0.07), they have a chance to move upward. Countries with RIVAPCs >0.07 will fail to maintain their industrial progress.

The findings are alarming, suggesting that the disparity between the Global North and the Global South will enlarge further in the future. Industrial development in the Global North will continue to prosper, while the industrial output in many countries in the Global South just cannot reach the global average. This calls for a re-examination of the industrialization policy in the Global South countries, and the governments should formulate pragmatic industrial policies to promote industrial development to close the gap between the two groups.

### Comparison Between Countries Based on Income Groups (World Bank Classification)

**Figure 9:**
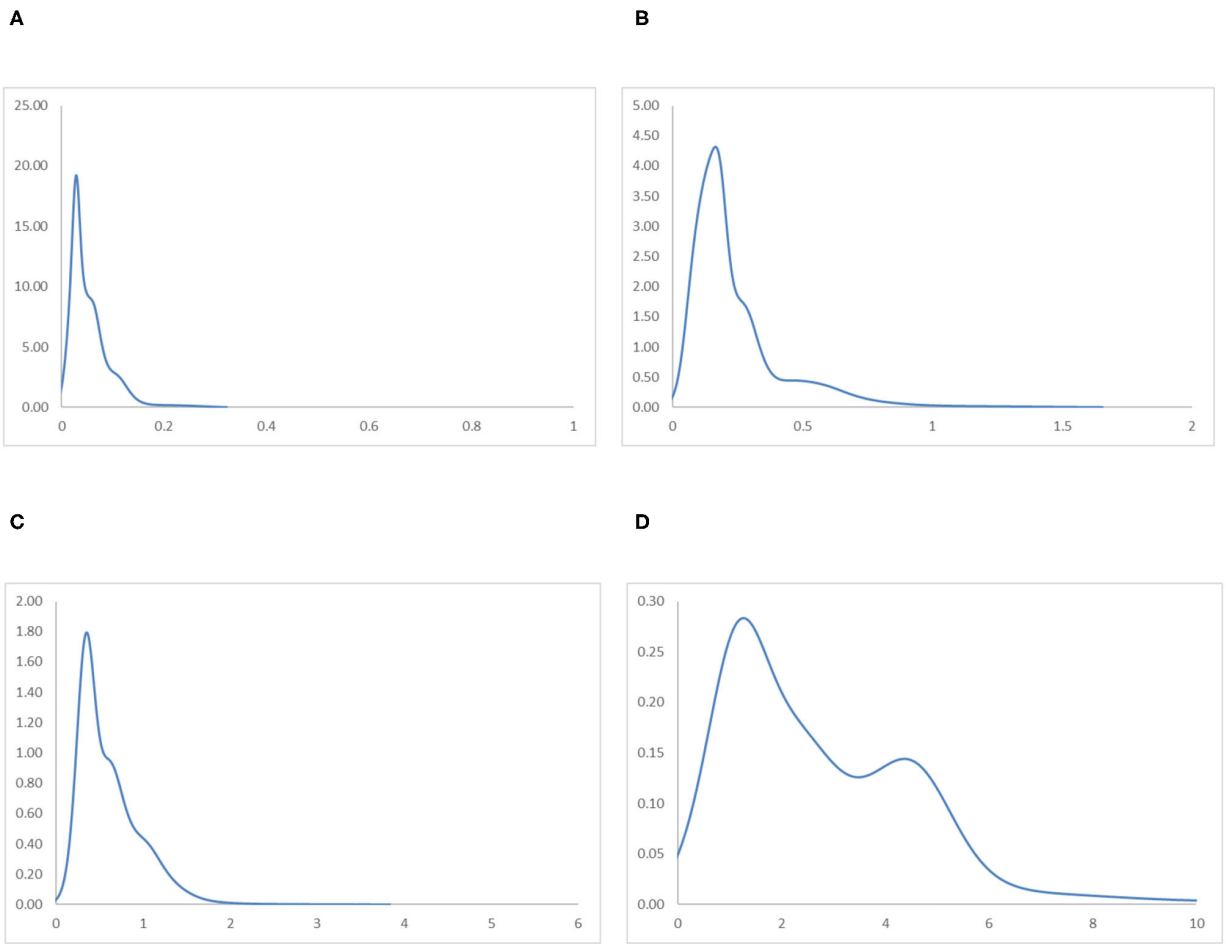
Ergodic distributions for the RIVAPC of countries for different income groups of countries with annual transitions. Source: authors' calculation. N.B. The vertical axis indicates the density of probability and the horizontal axis indicates RIVAPC values. **(A)** Low-Income Countries. **(B)** Lower-Middle Income Countries. **(C)** Upper-Middle Income Countries. **(D)** High-Income Countries.

**Figure 10:**
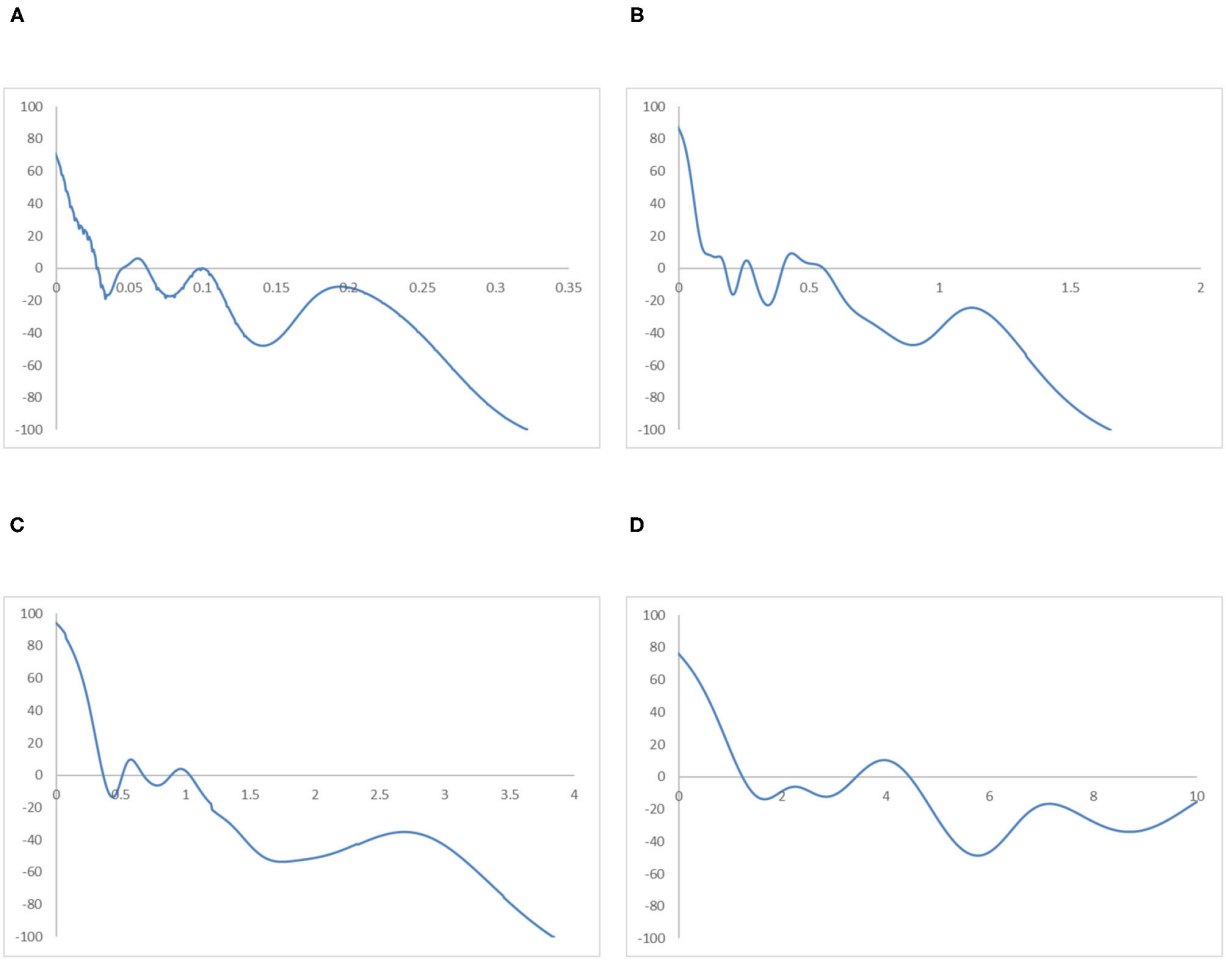
Mobility probability plot (MPP) for the RIVAPC of countries for different income groups of countries. Source: authors' calculation. N.B. The vertical axis indicates net upward mobility (%) and the horizontal axis indicates RIVAPC values. **(A)** Low-Income Countries. **(B)** Lower-Middle Income Countries. **(C)** Upper-Middle Income Countries. **(D)** High-Income Countries.

To avoid sporadic cases in the Global South region and to analyse the relationship between income and industrial development, the ergodic distributions for different income groups of countries are observed. Figures 9, 10 provide the ergodic distributions and the MPP for the four income groups, respectively.

It is apparent that for the low-income countries, the entire ergodic distribution in Figure 9A is compressed to the left of the global average, with the convergence clubs at the 0.03, 0.06, and 0.12 levels. Due to the distribution being highly concentrated, the peaks are visibly pronounced, and the convergence clubs are very close to each other. Looking at the MPP for the low-income countries in Figure 10A, only when the RIVAPC value is <0.03 do the countries have an upward-moving chance, while countries with RIVAPC values more than 0.03 have a negative net upward mobility. When the RIVAPC value reaches 0.3, the net upward mobility reaches −100. This implies that low-income countries cannot transit through the threshold of a RIVAPC value of 0.3. When countries reach a RIVAPC level of 0.3, a level less than half of the global average, the RIVAPC is going to decline, which will move the country downwards in the distribution. This implies that there might be a development trap in low-income countries. The situation is alarming, as industrialization is one of the major pathways for low-income countries to attain developed countries' living standards. The findings signify that poverty, if it exists, will persist unless there is outside interference.

For the lower-middle-income countries, in Figure 9B, although there are some observations around the global average, all the convergence clubs are below it, namely, 0.17, 0.29, and 0.59. Compared with low-income countries, the ergodic distribution of the lower-middle-income countries is more dispersed, and thus, countries will congregate around the three convergence clubs with a wider range, namely, 0.17 and 0.59. However, the third minor peak with the highest RIVAPC value of 0.59 is only slightly more than half of the global average. This demonstrates that the industrial development of lower-middle-income countries is far from encouraging. The MPP for the lower-middle-income countries in Figure 10B confirms the implications of the ergodic distribution. The first intersection point is situated at a very low RIVAPC level of 0.18, the second and the third range of positive net upward mobility are situated at RIVAPC levels of ~0.24–0.28, and 0.39–0.57, respectively, and the net upward mobility is −100 when the RIVAPC value reaches 1.6. In other words, when the low-middle-income countries have no or very low levels of industrialization, they have a better chance of having proper industrial development. When their level of industrialization is slightly more than half of the global average (i.e., a RIVAPC level of 0.57), they fail to maintain their development progress. Moreover, when the lower-middle-income countries have a RIVAPC level of 1.6, the net upward mobility reaches −100. It follows that when the countries reach a RIVAPC level of 1.6, it is going to have a decline in RIVAPC, which will bring the country downwards in the distribution. Although negative net upward mobility appears at a later stage of industrialization, as in the low-income countries, there seems to be a development trap in lower-middle-income countries. After reaching a slightly higher level of economic growth, the lower-middle-income countries fail to subsequently achieve further economic or industrial transformation required for sustainable development.

The situation is better in upper-middle-income countries. Three convergence clubs in Figure 9C can be observed. The first two peaks, 0.37 and 0.65, are below the global average. However, the third peak is just above the global average, which is level 1.12. Although the overall shape of the ergodic distribution for the upper-middle countries is quite similar to that in the low-income and lower-middle-income countries, they are not comparable as they are more dispersed and have a relatively higher average level of RIVAPC than the previous two distributions. Looking at the MPP in of Figure 10C for the upper-middle-income countries, positive net upward mobility can be seen within the range of RIVAPC values from 0.9 to 1.1, indicating that some countries are capable of maintaining sustainable development around the global average. Yet, as in the previous two income groups, a development trap can be found, as the net upward mobility is −100 when the RIVAPC value reaches 3.85. To avoid the upper-middle-income development trap, it is critical for upper-middle-income countries to reach and sustain a high rate of industrial development.

In high-income countries, the first peak of the ergodic distribution in Figure 9D appears at 1.37, while the second peak appears at 4.5. This indicates that many countries within the high-income group will have above the average industrial development and a few of them even have a production capacity that is 4.5 times higher than the rest of the world. Looking at its MPP in Figure 10D, the first intersection point appears at the RIVAPC level of 1.28, and the second intersection point is situated at the RIVAPC level of 3.4. These two intersection points give the two convergence clubs appearing in the ergodic distribution. Unlike the previous three MPPs for the low-income, lower-middle income, and upper-middle-income countries, no obvious development traps can be observed within the high-income countries group. The most negative net upward mobility is ~-50 when the RIVAPC reaches the 5.7 level.

Referring to the income groups evidence, this suggests that the primary peaks of the ergodic distribution for all income groups are far below the global mean with only one exception, the high-income group; suggesting that income level and industrial output might be related.

### Comparison Between Regions

**Figure 11:**
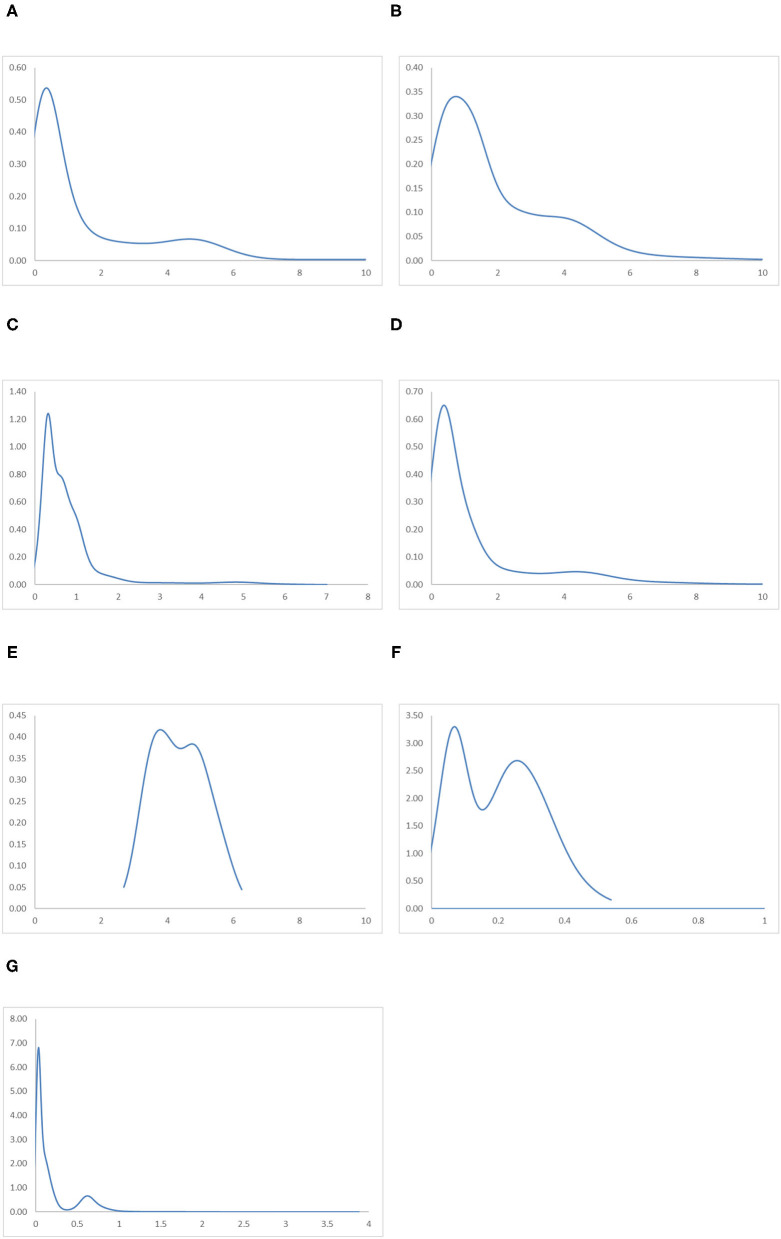
Ergodic distributions for the RIVAPC of countries for different geographical regions with annual transitions. Source: authors' calculation. N.B. The vertical axis indicates the density of probability and the horizontal axis indicates RIVAPC values. **(A)** East Asia and Pacific. **(B)** Europe and Central Asia. **(C)** Latin America and Caribbean. **(D)** The Middle East and North Africa. **(E)** North America. **(F)** South Asia. **(G)** Sub-Saharan Africa.

**Figure 12:**
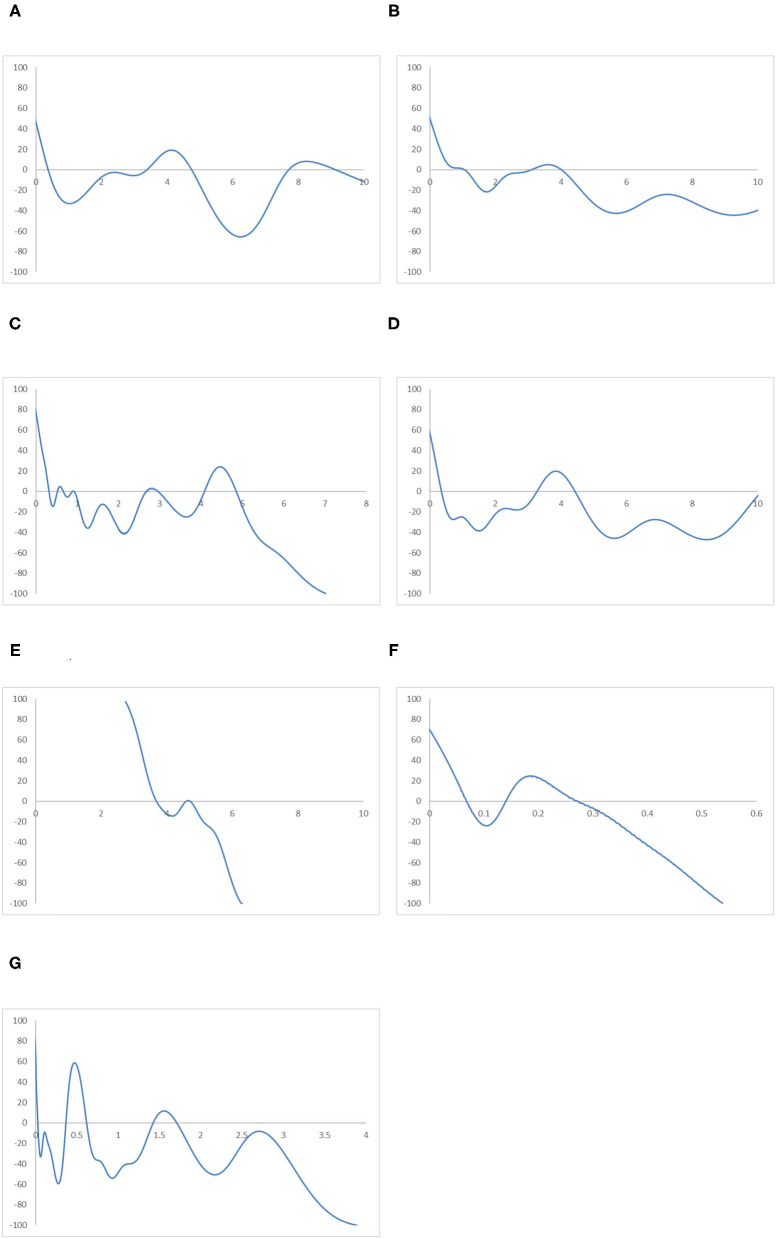
Mobility probability plot (MPP) for the RIVAPC of countries for different geographical regions. Source: authors' calculation. N.B. The vertical axis indicates net upward mobility (%) and the horizontal axis indicates RIVAPC values. **(A)** East Asia and Pacific. **(B)** Europe and Central Asia. **(C)** Latin America and Caribbean. **(D)** The Middle East and North Africa. **(E)** North America. **(F)** South Asia. **(G)** Sub-Saharan Africa.

Figure 11 shows the regional ergodic distributions and Figure 12 shows the MPP. The ergodic distributions and the MPP for the East Asia and Pacific region and the Middle East and North Africa region are quite similar. Within these two regions, it can be observed from their ergodic distribution that many countries cluster around RIVAPC levels around half of the global average, while a few countries cluster at RIVAPC levels around 4–5 times the global average. Their MPP shares similar momentum with the first and the second intersection points situated around the RIVAPC levels of 0.5 and 3.7, respectively. This implies that most countries will have output levels far below the mean, while a few will have an output level almost four times higher than the global average; this indicates that a high level of disparity appears in these regions.

A similar situation can be observed in the Europe and Central Asia region, apart from the fact that the first peak of the ergodic distribution in the region is situated at around a RIVAPC value of one; this indicates that most countries will converge to the global mean in this region. Thus, the region has done a relatively good job in maintaining its industrial growth around the global average. The MPP of the region confirms the implications of the ergodic distribution. The first intersection point appears around the RIVAPC value of one, and the second intersection point appears around the RIVAPC value of 3.2. These intersection points show that the primary and the secondary peaks, namely 0.9 and 4.1, appear in the ergodic distribution; this means that most of the countries will converge to the global mean, while a few will converge to a value 4 times higher than the global average. These findings are encouraging as the degree of disparity is tolerable as long as most countries within the regions maintain their industrial progress in line with the global average.

Unlike the previous three regions, the ergodic distribution in the Latin America and Caribbean region has four peaks, namely, 0.3, 0.6, 1.02, and 4.7. However, the second and the third peaks are not obvious; thus, most countries will converge to a RIVAPC value slightly >0.3. Almost the entire MPP of the region is situated below the horizontal axis after a RIVAPC value of one, which is the global average, with a positive region within the RIVAPC values of 4–5. This signifies that most of the countries in the region will have limited production capacity, while a small group of countries will enjoy an enormously high level of industrialization.

The ergodic distributions and the MPP for the North America and South Asia regions share similar shapes. However, they are incomparable, as the entire distribution for the South Asia region is located below a RIVAPC value of 0.6, while the entire distribution for North America is located above a RIVAPC value of 2. Comparing their MPP, the first and second intersection points for the North America region are 3.7 and 4.8, respectively, whereas the first and the second intersection points for the South Asia region are 0.02 and 0.37, respectively. The North America region is the only region where all countries have a RIVAPC more than the global average. In South Asia, most, if not all, countries within the region have a RIVAPC less than one. Indeed, the North America region represents the most industrialized region among the seven regions, whereas the South Asia region is the least industrialized region among the seven regions. Note that both regions suffer from development traps; the net upward mobility is −100 when the RIVAPC value reaches 6.3 and 0.53 in North America and South Asia, respectively. The development traps that appear in the South Asia region will hinder industrial development in the region, while development traps in the North America region will impede breakthroughs in the world industrial development.

The Sub-Saharan Africa region has the most compact ergodic distribution compared with the other regions, implying that the two peaks, 0.03 and 0.63, are very pronounced. Note that the primary peak of its ergodic distribution is just slightly higher than the South Asia region, which is the least industrialized region. This implies that most countries in this region will have very low industrial output. Note also that the net upward mobility value is −100 when the RIVAPC value reaches 3.88, and there is a positive net upward mobility region within the RIVAPC values of 1.41–1.72. Although the positive net upward mobility fails to translate into a third peak in the ergodic distribution, it indicates that there are few countries in the region enjoying a higher than the average production capacity.

Referring to the regional evidence, this suggests that the peaks of the ergodic distribution for most regions are far below the global mean with only two exceptions, North America and Europe and Central Asia. This means that convergence to the global mean is unattainable in almost all regions. Note also that convergence clubs can be observed in all regions, suggesting that disparity in industrial development at the regional level is universal. Attention must be paid to removing upward-moving obstacles for countries with low RIVAPC levels and negative net upward mobility.

## Conclusions and Implications

Many researchers have investigated the industrialization, industrial output and industrial development, little attention has been paid to whether differences in industrial development among different countries vanish over time, and if convergence can be realized. Most of the previous studies on convergence of industrial output or industrial development used single country's data and thus could not reveal many policy insights.

This paper examined the convergence patterns and dynamics of relative industrial value-added per capita in a global scale. It is the first study to investigate the convergence and dynamics of industrial value-added per capita in the world. With the consideration of heterogeneity in industrial value-added per capita, the results are useful to policy makers in identifying the key groups for priority interventions.

It was found that, in the all-countries analysis, among countries which had below-average industrial development, that is, those with a RIVAPC less than one, only those countries which had extremely low output levels (i.e., a RIVAPC <0.12) had a positive chance to move upward within the distribution. Conversely, countries with slightly higher output levels (i.e., a RIVAPC >0.12) cannot maintain their progress in industrial development.

In the comparison between the Global North and the Global South, it was found that only countries in the Global South have no industrialization at all (those countries with a RIVAPC value <0.07), they have a chance to move upward. Countries with RIVAPCs >0.07 will fail to maintain their industrial progress.

In terms of Income Groups (World Bank Classification), it was found that the primary peaks of the ergodic distribution for all income groups are far below the global mean with only one exception, the high-income group; suggesting that income level and industrial output might be related. This study also found that the peaks of the ergodic distribution for most regions are far below the global mean with only two exceptions, North America and Europe and Central Asia.

Based on the findings, the following policy implications can be drawn: first, policy makers need to pay more attention to the countries that have an RIVAPC level below global average. For those countries that will converge to RIVAPC of <0.5, industrial development planning and policy measures need to be implemented. Governments should provide more support to those underdeveloped countries in a more targeted manner through technical co-operation with developing countries.

The results of distribution of the Relative Industrial Value-Added per Capita (RIVAPC) of global countries provide policy makers with reliable reference to improve global industrial development strategies by prioritizing supportive policies across the countries. In order to eliminate the inequalities in industrial development among regions, United Nations and the governments should provide more support in the Latin America region, Caribbean region, South Asia region and the Sub-Saharan Africa region.

## Data Availability Statement

Publicly available datasets were analyzed in this study. This data can be found here: https://databank.worldbank.org/reports.aspx?source=global-financial-development&Type=METADATA&~preview=on#.

## Author Contributions

NM, WS, TH, and TC: conceptualization, data curation, methodology, software, visualization, writing–original draft preparation, and writing–review and editing. All authors contributed to the article and approved the submitted version.

## Funding

This work was supported by the Hainan College of Economics and Business (Project Reference Number: hnjmk2021301). This work was supported by the Faculty Development Scheme (FDS) of the Research Grants Council of Hong Kong, China (Project Reference Number: UGC/FDS14/B19/16).

## Conflict of Interest

The authors declare that the research was conducted in the absence of any commercial or financial relationships that could be construed as a potential conflict of interest.

## Publisher's Note

All claims expressed in this article are solely those of the authors and do not necessarily represent those of their affiliated organizations, or those of the publisher, the editors and the reviewers. Any product that may be evaluated in this article, or claim that may be made by its manufacturer, is not guaranteed or endorsed by the publisher.
